# Central sensitization in knee osteoarthritis and fibromyalgia: Beyond depression and anxiety

**DOI:** 10.1371/journal.pone.0225836

**Published:** 2019-12-05

**Authors:** Marina López-Ruiz, Josep Maria Losilla, Jordi Monfort, Mariona Portell, Teresa Gutiérrez, Violant Poca, Ferran Garcia-Fructuoso, Jone Llorente, Alba Garcia-Fontanals, Joan Deus

**Affiliations:** 1 Service of Psychiatry and Psychology, HM-Sant Jordi Clinic, Barcelona, Spain; 2 Department of Methodology, Faculty of Psychology of Autonomous University of Barcelona, Cerdanyola del Vallés, Spain; 3 Rheumatology Service, Hospital del Mar, Barcelona, Spain; 4 Department of Clinical and Health Psychology, Faculty of Psychology of Autonomous University of Barcelona, Cerdanyola del Vallés, Spain; 5 Rheumatology Service, Institute Ferran of Rheumatology (IFR), Barcelona, Spain; 6 Benito Menni Complex Assistencial en Salut Mental, Sant Boi de Llobregat, Spain; Medical University of Vienna, AUSTRIA

## Abstract

**Objectives:**

To determine the psychopathological profile of patients with central sensitization (CS) in a sample of knee osteoarthritis, with and without CS, and fibromyalgia, and to compare their psychopathological profiles.

**Methods:**

The final sample consists of 19 patients with osteoarthritis and CS (mean 66.37 years ± 8.77), 41 osteoarthritis patients without CS (mean 66.8 ± 7.39 years), 47 fibromyalgia patients (mean 46.47 years ± 7.92) and 26 control subjects (mean 51.56 years ± 11.41). The psychopathological profile was evaluated with the Millon Multiaxial Clinical Inventory.

**Results:**

The average score of MCMI-III reflect higher scores in the fibromyalgia and osteoarthritis-CS groups. Patients with osteoarthritis-CS are more likely to report larger scores in Borderline and Major Depression scales. Fibromyalgia patients are more likely to report more increased scores in Somatoform and Major Depression, versus osteoarthritis-CS group. Fibromyalgia patients versus osteoarthritis without CS are more likely to report higher scores in Schizoid, Depression, Histrionic, Sadistic, Borderline, Somatoform, Posttraumatic Stress Disorder and Major Depression scales.

**Discussion:**

Patients with CS have less differences in their psychopathological profiles as well as in both osteoarthritis groups and greatest differences are obtained between the fibromyalgia and osteoarthritis without CS, so perhaps presence of CS is the key to differentiate those groups and not chronic pain. An exhaustive assessment brings more accurate psychopathological profiles, thus better psychological treatment could be applied.

## Introduction

Musculoskeletal disorders are one of the most common causes of disability and incapacity due to pain. Two diseases where chronic pain is typically present are osteoarthritis (OA), knee OA as the most frequent type, and fibromyalgia (FM). About 10% of people aged over 55 years have painful disabling knee OA of whom one quarter are severely disabled [1]. The prevalence of FM is 2.5% in Europe with a female-to-male ratio of 3:1. [2].

Knee OA is a common chronic condition causing disabling symptoms, such as joint pain, physical and psychological dysfunction, and reduced quality of life (QoL) [3]. Psychological distress, including depression, depressed mood or anxiety has been associated with higher levels of pain in OA patients [4]. A 7-year follow-up study linked OA to affective disease (depression and bipolar disorder), personality disorders and substance abuse [5]. Knee OA pain is likely a heterogeneous, multifactorial phenomenon that involves not only the OA disease process but also elements specific to patient psychology and pain neurophysiology [6]. In fact, there is an emerging consensus that the degree of knee pain and disability symptoms in OA patients seems to rest upon various factors, including structural damage, peripheral and central pain processing mechanisms and psychosocial factors among others (obesity, culture and demographic) [7]. Despite the evidence which relate psychological factors and OA chronic pain, the Osteoarthitis Initiative [8] and Stubbs et al. review [9] found unclear outcomes in the explanation of this relationship. Perhaps, the heterogeneity of OA chronic pain, constant and intermittent pain, with or without a neuropathic component and with or without central sensitization (CS) [10], may illuminate these contradictions.

FM is a chronic disease characterized by widespread musculoskeletal pain and hyperalgesia after digital pressure in at least 11 of 18 tender points [11]. Since 2010, the criteria also recognize the importance of a quantitative measure of widespread pain, the widespread pain index, incorporating key fibromyalgia symptoms and providing severity scales to measure the extent of widespread pain and symptom severity [12]. FM is considered the most typical disease of CS syndromes [13], although, some authors consider there are some FM patients without CS due to the clinical heterogeneity of those patients [14]. CS As in OA, in FM there has been found a relationship between it and affective symptomatology [15], where FM patients have enhanced scores in depression and anxiety questionnaires [16] as well as increased presence of psychopathology [16]. Emotional and affective symptoms are one of the best contributing factor which better predict FM impact, and level of health perception [16]. Furthermore, the presence of psychopathological diseases may influence the impact of FM in daily activities [17], health state [16], and pain intensity [18]. It also has been found that the relationship between depression, mental stress and anxiety, and FM is bidirectional [19]. However, some investigations show that not all FM patients suffer psychopathology [20], so they are a heterogeneous group [21]. There are few studies related to FM pain where results are contradictory. Some of them show no relation neither between emotional symptoms and FM pain [18], nor with induced pain [22,23]. In contrast, other studies found relation between pain intensity and emotional symptoms, like anxiety, depression and anger [18].

On one hand, OA and FM patients share suffering chronic pain. On the other, they also share the presence of sensitization of Central Nervous System (CNS). CS is a phenomenon that appears normally when there is pain. When it is acute, the sensitization is so. However, in chronic pain patients, sensitization remains present after nociception has resolved [24]. CS manifests as pain hypersensitivity with anatomically spread hyperalgesia (to feel grater pain than the stimulus causing this pain), enhanced temporal summation of pain after repeated stimulation [25], and allodynia (to feel pain after normally non-painful stimulation such as touch). CS in OA patients include extended and remote areas of hyperalgesia from the affected joint, a loss of descending pain inhibitory mechanisms and an increase of temporal summation and spatial summation [26]. In knee OA patients, Lluch et al. [27] found expanded distribution of pain (by shading the painful area in pain drawings) was correlated with some measures of CS. Wood et al. [28] found that people with knee OA reporting enlarged areas of pain had more persistent and severe pain and higher anxiety levels, which also was interpreted as reflecting altered central pain processing mechanisms. In FM patients, CS may be the characteristic feature of the disorder. In OA, not all patients have CS, but only a subgroup. This may be a possible explanation why there is no congruence between radiological findings and perception of pain [29].

Even it is known that CS is a relevant phenomenon in the explanation of chronic pain, in our opinion, there are very few investigations regarding emotional and affective symptoms or disease impact or functional disabilities. George et al. [30] found that pain related fear contributed to hyperalgesia, while pain catastrophizing contributed to temporal summation, two of the CS features. Imamura et al. [31] studied the implications of the presence of CS and found that its presence, measured by evaluated superficial and deep hyperalgesia by assessing pressure pain threshold, correlates with lower pressure pain thresholds, larger intensity of knee pain (assessed by a visual analogic scale), reduction of functional capacity (measured by WOMAC) and poorer QoL (assessed by SF-36) in patients with knee OA. As said before, Wood et al. [28] also found knee OA patients with biggest pain areas show higher levels of anxiety. As CS is common in various pathologies, including OA and FM, maybe we also need a new perspective addressed to study central sensitization syndrome [32], rather than the disease itself, to know if there are similar characteristics in medical and psychological aspects and offer a better treatment.

So, the aims of this study are to determine the psychopathological profile of OA patients with and without CS, and to compare these psychopathological profiles with that of FM patients.

## Materials and methods

This study was carried out at the Rheumatology Department of both, Hospital del Mar and Hospital CIMA-Sanitas, in Barcelona (Spain). It was approved by the Local Ethics Committee and was in compliance with the Helsinki Declaration.

### Participants

The patients were selected by a senior rheumatologist and a senior psychologist during a period of 18 months. The initial sample was composed by 90 patients with osteoarthritis diagnosis and 150 with fibromyalgia diagnosis at the Rheumatology Department of Hospital del Mar and Hospital CIMA Sanitas, in Barcelona. There were also 35 healthy controls (C). Patients with OA were separated into 2 sub-groups: (a) presence of clinical CS (OA-CS, *n* = 28) and (b) absence of CS (OA-noCS, *n* = 62). It is defined by the presence of both spreading sensitization and temporal summation to repeated pressure pain stimulation [33].

The main inclusion criteria for OA patients were: (1) radiological and clinical diagnosis of knee OA based on American College of Rheumatology (ACR) criteria [34] affecting at least one knee of a minimum of 3 months in symptom duration prior to screening; (2) male or female (non-childbearing potential) at least 45 years old; (3) a minimum of 4 out of 10 on the numerical rating scale (Brief Pain Inventory, item 5) at screening and/or a requirement for the use of an analgesic for the knee pain. Regarding the CS subgroups, the inclusion criteria for presence of OA-CS group were: (1) clinical evidence of pain or altered sensations spread beyond the knee joint by manual palpation in baseline rheumatologist assessment; (2) at least 3 tender points in the extended version of the Arendt-Nielsen [33] peripatellar map (excluding points 3, 7 and 8, which are part of the joint itself) -a tender point is defined as a point showing a mechanical pressure pain threshold below 4 kg/cm^2^ [11]; (3) pain score of 4 points or more in an 11-point verbal scale during 2-second 4 kg/cm^2^ pressure stimulation on the anterior surface of the tibial bone; (4) presence of temporal summation (increase of more than 1 point in an 11-point verbal scale after 10 repeated pressure stimulation at 1 second inter-stimulus intervals) on the most sensitive site of the peripatellar region [33].

The main inclusion criteria for FM patients were: (1) a diagnosis of FM following the ACR criteria (1990); (2) history of widespread non-articular pain with insidious onset over 3 months; (3) one year minimum of disease evolution; (4) presence of CS; (5) absence of comorbid Chronic Fatigue Syndrome.

Inclusion criteria for control group selection were as follows: no history of rheumatic disorder, no history of functional pain or physical widespread pain, no history of Axis I or II psychiatric illness, and no history of neurological disease. In all groups, patients with a history of psychotic disorder or substance abuse, patients with a history or diagnosis of personality disorders and patients with a history of neuropathic pain were not included. The participants signed informed consent to accept the conditions of the study. This written consent was corresponding to a larger protocol which included the present study. We have used a systematic and rigorous process that allows us to ensure the sample fulfill strictly all the inclusion/exclusion criteria, so the sample is very well delimited.

The final sample was made up of 19 patients with OA and CS (OA-CS) aged between 44 and 81 (mean 66.37 yrs±8.77), 41 with OA without CS (OA-no CS) aged between 46 and 79 (mean 66.8 yrs±7.39), 47 FM patients aged between 32 and 63 (mean 46.47 yrs±7.9) and 26 participants in the control group aged between 50 and 77 (mean of 51.56 yrs±11.41). Most relevant characteristics of the sample are shown in Table 1.

**Table 1:** Sociodemographic and clinical characteristics of the sample.

	Groups				
Factors	OA	OA-CS	OA-noCS	FM	Control
N	60	19	41	47	26
Age $\bar{x}$(SD)	66.67 (7.78)	66.37 (8.77)	66.8 (7.39)	46.47 (7.92)	62.92 (7.39)
Gender (women %)	71.7	84.2	65.9	100	59.3
Months after diagnosis $\bar{x}$(SD)	55.28 (63.34)	50.58 (54.09)	57.46 (67.71)	84.38 (54.14)	-
Educational level (%)					
Uneducated	6.7	10.5	4.9	0	0
Primary education	21.6	26.3	19.5	10.6	26.9
Secondary education	15	10.4	17.1	21.3	12.1
General education	5	10.5	2.4	12.8	7.4
Vocational education and training and/or Higher education	31.7	36.8	29.3	17	30.6
Bachelor and/or University Degree	20	5.3	26.8	38.3	23
Drug use (%)					
Painkiller	50	16	34	16	0
Non-steroidal Anti-inflammatory drugs (NSAIDs)	51	16	35	17	2
Antiepileptic drugs	2	0	2	4	3
Antidepressant drugs	14.9	15.8	19.5	85.7	7.4
Questionnaires (pain and cognition)					
WOMAC pain $\bar{x}$(SD)	7.45 (2.85)	8.74 (3.1)	6.85 (2.55)	-	-
WOMAC shiftiness $\bar{x}$(SD)	2.17 (1.85)	2.58 (2.58)	1.98 (1.73)	-	-
WOMAC function $\bar{x}$(SD)	20.83 (10.4)	23.74 (11.32)	19.49 9.79)	-	-
WOMAC total $\bar{x}$(SD)	30.45 (13.13)	35.05 (13.6)	28.32 (12.05)	-	-
FIQ total $\bar{x}$(SD)	-	-	-	65.99 (14.01)	-
Mini Mental	27.61 (2.68)	26.82 (3.17)	28 (2.36)	-	27.5 (2.45)

OA: osteoarthritis, OA-CS: osteoarthritis with Central Sensitization, OA-noCS: osteoarthritis without Central Sensitization, FM: fibromyalgia, SD: standard deviation, FIQ: fibromyalgia impact questionnaire, Mini-Mental: OA patients carried out cognitive screening because of their advanced age and the possibility of cognitive impairment. FM patients did not need it due to their age and study objectives.

### Procedure

All patients went first to rheumatologic visit where they were selected and, after check the inclusion/exclusion criteria and the will to participate, they were also enrolled. During the same week, the patient went to the visit of psychological assessment, always done by the same clinical psychologist, which last 2 hours, approximately. If necessary, we visit again the patient in case of too much fatigue that may influence the responses. The completed protocol is wide and in the present research we only use some parts of it.

### Assessment

Millon Clinical Multiaxial Inventory, MCMI-III, Spanish version [35]. The MCMI-III is a self-reported measure of psychopathology. It consists of 175 true–false questions that measure 4 validity indices, 11 clinical personality patterns, 3 severe personality disorders (14 scales of Axis II), 7 clinical syndromes and 3 severe clinical syndromes (10 clinical syndromes of Axis I). The different scales correspond to DSM-IV nosology [35]. A cut-off score of 75 or more for each of the 10 clinical syndrome scales indicates a probable Axis I diagnosis, as well as presence of personality traits clinically significant on the 14 scales of Axis II. In the original version Cronbach’s alpha scores between 0.66 and 0.90 and in the Spanish version between 0.65 and 0.88 [35]. We choose this questionnaire because it has some interesting characteristics: 1) it is much shorter than comparable instruments, minimizing fatigue; 2) scale elevations and configurations can be used to suggest specific patient diagnoses and clinical dynamics; 3) profiles based on all clinical scales may be interpreted to show the interaction between long-standing characterological patterns and the distinctive clinical symptoms currently manifest; and 4) reflect the DSM distinction between Axis I and Axis II.

Fibromyalgia Impact Questionnaire (FIQ) [36]. It is a self-report questionnaire with 10 items. This instrument measures the impact of FM on functional capacity and quality of life. FIQ scores range from 0 to 100, where 0 indicates the best functional capacity and quality of life and 100 the poorest. We used the Spanish version which the intraclass correlation coefficient for the total S-FIQ was 0.81 and retained the methodological properties of the original version [37].

Western Ontario and McMaster Universities Osteoarthritis Index (WOMAC) [38]. It is a self-reported health status questionnaire which contains three dimensions: pain, stiffness and function. These dimensions score range of 0 to 20, 0 to 8, and 0 to 68, respectively, with higher scores indicating more pain, stiffness, and reduced physical function. It also has a visual analogue scale (VAS). The psychometric properties of the Spanish version [39] are regarding construct validity, correlations ranged from 0.30 to 0.84 for VAS and 0.27 to 0.77 for Pain, Stiffness and Difficulty subscales. In regards of internal consistency, the Cronbach’s alpha coefficients ranged from 0.71 to 0.97 for the VAS and 0.64 to 0.95 for the 3 subscales. In test–retest reliability, the correlation coefficients ranged from 0.36 to 0.76 for VAS and 0.34 to 0.52 for 3 subscales [40].

### Statistical analysis

We carried out a descriptive analysis to know if there are differences between the 4 groups (OA-CS, OA-noCS, FM and C) in MCMI-III scores, and logistic regression analyses to investigate the most characteristic MCMI-III psychopathological profile of those 4 groups. The results of the association between MCMI-III scores and the four binary responses investigated (OA patients *versus* controls, OA-CS *versus* OA-noCS patients, OA-CS *versus* FM patients, and OA-noCS *versus* FM patients) are presented as non-linear logistic regression coefficients (OR_adj_) with the corresponding 95% confidence intervals (95% CI), and P-values (P). We include also the transformation of OR_adj_ into a percentage of change ((OR_adj_—1) * 100) to facilitate its interpretation. The initial logistic regression models also included gender, age, academic level and cognitive screening as potentials confounders. Regression backward model selection was conducted, fitted using IBM SPSS Statistics package (IBM Corp. Released 2011. IBM SPSS Statistics for Windows, Version 20.0. Armonk, NY: IBM Corp.).

## Results

After the analysis to investigate the psychopathological profile of OA-CS, OA-noCS, FM and C we found the following results.

**Fig 1:**
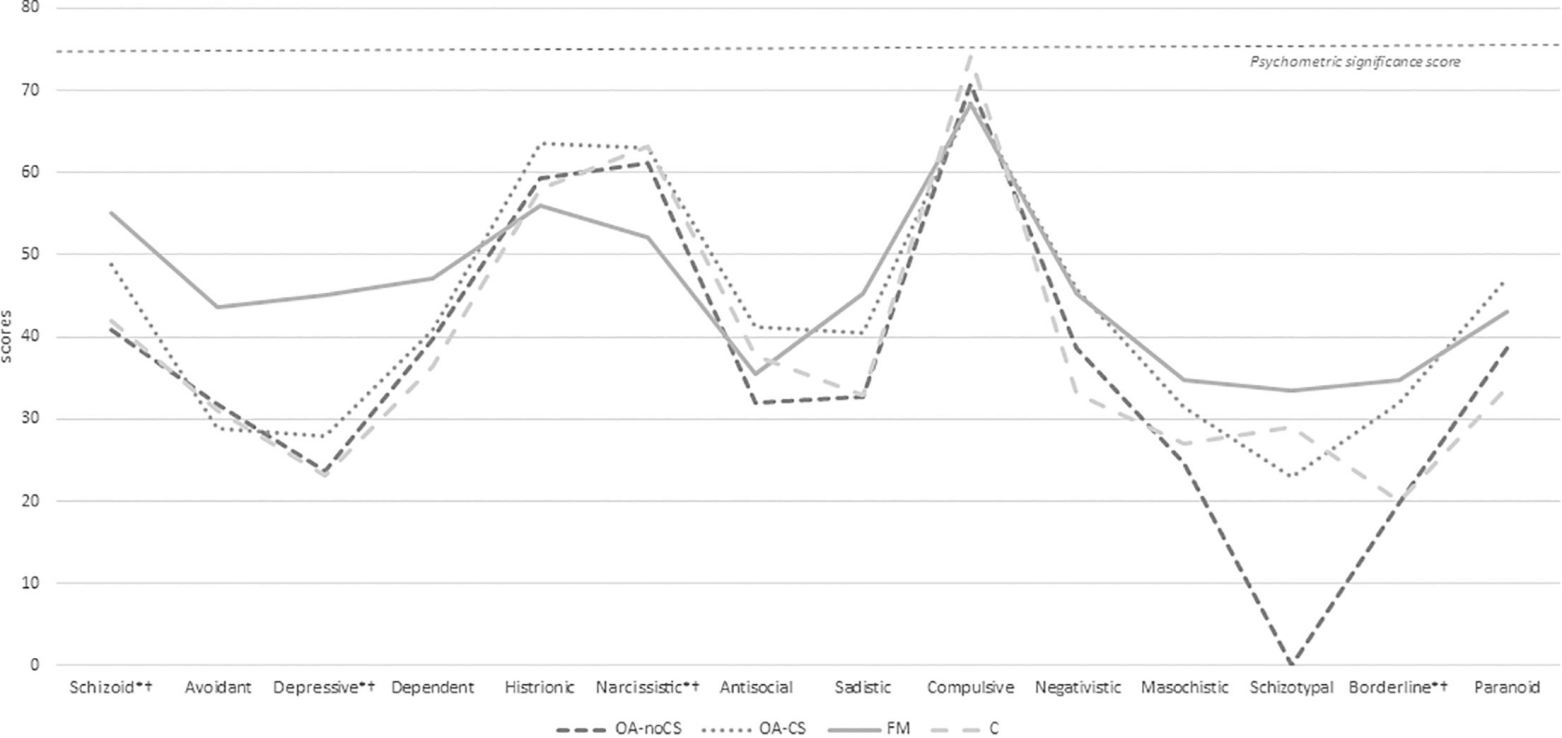
Mean scores in MCMI-III for clinical personality patterns and severe personality pathology. *statistical differences between groups. †: FM differences, versus OA-noCS, after Scheffé multiple comparisons test. OA-noCS: osteoarthritis without Central Sensitization, OA-CS: osteoarthritis with Central Sensitization, FM: fibromyalgia, C: control group.

**Fig 2:**
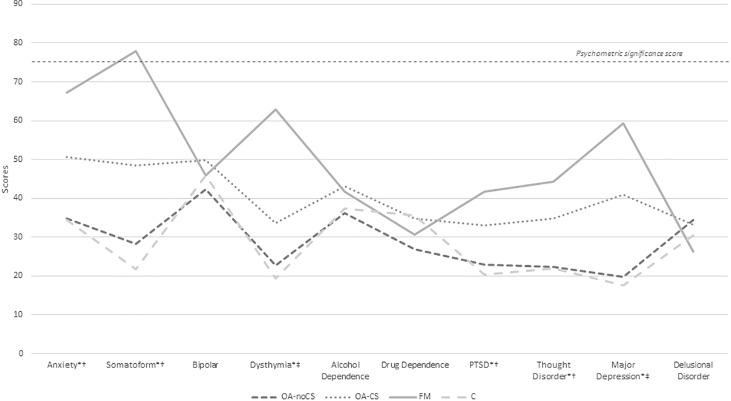
Mean scores in MCMI-III for clinical syndrome and severe clinical syndrome. *statistical differences between groups. †: FM differences, versus OA-noCS, after Scheffé multiple comparisons test. ‡: FM differences, versus OA group, after Scheffé multiple comparisons test. OA-noCS: osteoarthritis without Central Sensitization, OA-CS: osteoarthritis with Central Sensitization, FM: fibromyalgia, C: control group.

Figs 1 and 2 show the mean scores of MCMI-III subscales, where, under an overall look, we can see the largest scores are reported by FM patients and OA-CS patients, mainly. Table 2 shows the MCMI-III scores in all subscales.

**Table 2:** Sample size, mean scores and standard deviation of patients with osteoarthritis, with and without central sensitization, fibromyalgia and control subjects, in MCMI-III.

	Min-Max					$\bar{x}$ (SD)				
	OA (N = 44)	OA-CS (N = 14)	OA-noCS (N = 30)	FM (N = 43)	C (N = 21)	OA	OA-CS	OA-noCS	FM	C
Schizoid	0–71	14–71	0–69	17–85	9–74	43,41 (19,754)	48,71 (18,507)	40,93 (20,127)	54,98 (16,162)	42,00 (21,679)
Avoidant	0–80	0–74	0–80	0–78	0–76	30,84 (25,676)	28,86 (26,085)	31,77 (25,879)	43,53 (24,641)	31,05 (25,621)
Depressive	0–81	0–81	0–76	0–81	0–73	25,05 (22,823)	27,93 (29,056)	23,70 (19,698)	45,07 (24,803)	23,10 (24,041)
Dependent	0–80	0–69	0–80	6–91	0–76	40,07 (21,868)	40,86 (22,027)	39,70 (22,161)	47,07 (23,568)	36,43 (19,600)
Histrionic	30–93	37–78	30–93	5–83	23–88	60,66 (15,595)	63,57 (12,017)	59,30 (17,026)	56,05 (19,998)	58,05 (17,659)
Narcissistic	16–81	24–81	16–80	6–71	47–79	61,73 (14,631)	63,00 (12,782)	61,13 (15,589)	52,02 (17,204)	63,19 (8,116)
Antisocial	0–68	0–65	0–68	0–67	0–63	34,91 (21,724)	41,21 (21,420)	31,97 (21,586)	35,56 (21,203)	37,71 (19,368)
Sadistic	0–69	0–69	0–64	0–75	0–67	35,16 (23,801)	40,50 (26,055)	32,67 (22,704)	45,26 (21,036)	33,00 (24,423)
Compulsive	49–93	49–88	49–93	33–98	54–93	70,11 (13,356)	68,57 (13,013)	70,83 (13,671)	68,33 (13,685)	74,10 (11,273)
Passive-aggressive	0–77	11–77	0–69	5–75	0–67	40,89 (20,669)	45,86 (22,003)	38,57 (19,974)	45,35 (20,251)	33,05 (23,581)
Masochistic	0–64	0–64	0–61	0–68	0–64	26,75 (21,465)	31,50 (23,774)	24,53 (20,343)	34,81 (23,143)	27,10 (23,537)
Schizotypal	0–61	0–60	0–61	0–64	0–69	20,91 (23,589)	22,86 (26,038)	20,00 (22,770)	33,56 (26,749)	29,00 (27,595)
Borderline	0–62	7–62	0–62	0–75	0–66	23,70 (19,022)	31,93 (18,862)	19,87 (18,143)	34,74 (21,959)	20,10 (19,372)
Paranoid	0–93	0–93	0–77	0–74	0–100	41,30 (25,480)	47,07 (26,459)	38,60 (25,004)	43,09 (22,241)	33,86 (32,714)
Anxiety	0–94	15–89	0–94	9–107	0–90	39,84 (30,891)	50,64 (23,957)	34,80 (32,788)	67,33 (29,635)	34,33 (35,132)
Somatoform	0–97	0–75	0–97	12–115	0–75	34,68 (28,944)	48,50 (25,022)	28,23 (28,746)	77,91 (21,962)	21,67 (22,999)
Bipolar	0–78	20–75	0–78	0–82	0–82	44,75 (21,737)	49,86 (18,716)	42,37 (22,916)	45,95 (21,740)	45,86 (24,818)
Dysthymia	0–82	0–82	0–78	0–111	0–75	26,27 (25,589)	33,64 (24,806)	22,83 (25,625)	62,86 (29,598)	19,48 (24,200)
Alcohol dependence	0–69	0–66	0–69	0–66	1–66	38,45 (24,145)	43,21 (22,635)	36,23 (24,874)	41,77 (21,940)	37,29 (21,905)
Drug dependence	0–65	0–63	0–65	0–62	0–65	29,45 (22,378)	34,86 (20,369)	26,93 (23,149)	30,63 (24,690)	35,62 (25,011)
Posttraumatic stress disorder	0–68	0–67	0–68	0–71	0–69	26,18 (23,649)	33,07 (22,666)	22,97 (23,777)	41,98 (22,775)	20,43 (22,462)
Thought disorder	0–82	0–64	0–82	0–80	0–87	26,34 (24,173)	34,86 (25,946)	22,37 (22,656)	44,26 (24,284)	21,86 (27,266)
Major depression	0–95	0–77	0–95	0–100	0–70	26,57 (26,207)	41,00 (23,830)	19,83 (24,826)	59,37 (22,740)	17,57 (20,805)
Delusional disorder	0–89	0–89	0–80	0–69	0–93	33.59 (32,198)	33.14 (34,732)	34.33 (31.559)	31,325 (31.325)	30,48 (35,942)

OA: osteoarthritis; OA-CS: osteoarthritis patients with Central Sensitization; OA-noCS: osteoarthritis patients with Central Sensitization; FM: fibromyalgia patients; C: control subjects; Min: minimum score; Max: maximum score; SD: standard deviation. Sample size: some patients were excluded due to validity scales.

Table 3 condenses the percentages of cases with a PREV score higher than 75, which means psychometric significance. We can see a positive and significant association between Anxiety, Somatoform, Dysthymia and Major depression scales and the group of patients.

**Table 3:** Percentage of cases that show psychometric significance (PREV≥75) in OA and FM patients and control group.

	% OA-CS (N = 14)	% OA-noCS (N = 30)	% FM (N = 43)	% C (N = 21)	X^2^	*p*
Cut-off point PREV≥75						
Clinical Personality Patterns scales						
*Schizoid*	*0*	*0*	*4*.*7*	*0*	*3*.*08*	.*379*
*Avoidant*	*0*	*10*	*11*.*6*	*4*.*8*	*2*.*343*	.*504*
*Depressive*	*14*.*3*	*3*.*3*	*14*	*0*	*5*.*318*	.*150*
*Dependent*	*0*	*6*.*7*	*7*	*4*.*8*	*1*.*085*	.*781*
*Histrionic*	*28*.*6*	*16*.*7*	*18*.*6*	*19*	.*914*	.*822*
*Narcissistic*	*14*.*3*	*16*.*7*	*0*	*9*.*5*	*7*.*325*	.*062*
*Antisocial*	*0*	*0*	*0*	*0*	*-*	*-*
*Sadistic*	*0*	*0*	*2*.*3*	*0*	*1*.*526*	.*676*
*Compulsive*	*35*.*7*	*46*.*7*	*32*.*6*	*42*.*9*	*1*.*687*	.*640*
*Negativistic*	*7*.*1*	*0*	*2*.*3*	*0*	*3*.*172*	.*366*
*Masochistic*	*0*	*0*	*0*	*0*	*-*	*-*
Severe Personality Pathology scales						
*Schizotypal*	*0*	*0*	*0*	*0*	*-*	*-*
*Borderline*	*0*	*0*	*2*.*3*	*0*	*1*.*526*	.*676*
*Paranoid*	*14*.*3*	*3*.*3*	*0*	*9*.*5*	*6*.*297*	.*098*
Clinical Syndrome Scales						
Anxiety	21.4	23.3	51.2	19	10.323	.016
Somatoform	14.3	6.7	69.8	0	49.352	.000
*Bipolar*	*14*.*3*	*6*.*7*	*7*	*9*.*5*	.*901*	.*825*
Dysthymia	7.1	6.7	51.2	0	31.892	.000
*Alcohol Dependence*	*0*	*0*	*0*	*0*	*-*	*-*
*Drug Dependence*	*0*	*0*	*0*	*0*	*-*	*-*
*PTSD*	*0*	*0*	*0*	*0*	*-*	*-*
Severe Clinical Syndrome scales						
*Thought Disorder*	*0*	*3*.*3*	*4*.*2*	*4*.*8*	.*724*	.*868*
Major Depression	7.1	3.3	23.3	0	11.107	.011
*Delusional Disorder*	*7*.*1*	*3*.*3*	*0*	*14*.*3*	*6*.*837*	.*077*

OA: osteoarthritis; OA-CS: osteoarthritis patients with Central Sensitization; OA-noCS: osteoarthritis patients with Central Sensitization; FM: fibromyalgia patients; C: control subjects; PTSD: Posttraumatic Stress Disorder.

### OA patients versus controls

Table 4 summarizes the results of the logistic regression models that investigate the differential MCMI-III psychopathological profiles of OA patients *versus* C. OA patients differ from C in 4 clinical personality patterns, where OA patients are more likely to report higher scores in Histrionic (increase in ORadj between 2.3% and 24.6%) and Passive-aggressive (increase in ORadj between 2.4% and 17.2%) subscales, and lower scores in Antisocial (decrease in ORadj between 0.8% and 11.6%) and Compulsive (decrease in ORadj between 1.4% and 16.6%) subscales. Regarding severe personality pathology OA patients are more likely to report lower scores in Schizotypal (decrease in ORadj between 0.3% and 6%) subscale. The clinical syndrome which was different is Somatoform subscale, where OA patients are more likely to show higher scores (increase in ORadj between 0.3% and 7.6%).

**Table 4:** Differential MCMI-III psychopathological profiles of *OA patients versus controls*.

OA *vs* C				
		OR_adj_ (95% CI)	(OR_adj_—1) * 100	*p*
Clinical Personality Patterns	His	1.129 (1.023–1.246)	12.9% (2.3% − 24.6%)	.015
	Ant	.937 (.884 –.992)	-6.3% (-.8% − -11.6%)	.027
	Com	.907 (.834 –.986)	-9.3% (-1.4% − -16.6%)	.021
	Pas	1.095 (1.024–1.172)	9.5% (2.4% − 17.2%)	.008
Severe Personality Pathology	Schz	.968 (.94 –.997)	-3.2 (-.3%–-6%)	.032
Clinical Syndromes	Som	1.039% (1.003%– 1.076%)	3.9% (.3%– 7.6%)	.033
Severe Clinical Syndromes	*MDpr*	*1*.*039 (*.*998%– 1*.*081%)*	*3*.*9% (-*.*2%– 8*.*11%)*	.*060*

Logistic regression adjusted Odds ratio (OR_adj_), 95% confidence intervals (95% CI) and p-values (*p*). (OR_adj_—1) * 100 shows percentage of change per unit. The initial model included gender, age, academic level and cognitive screening, OA: osteoarthritis; C: control subjects; His: Histrionic; Ant: antisocial; Com: compulsive; Pas: passive-aggressive; Schz: schizotypal; Som: somatoform; MDpr: major depression.

### OA patients with CS versus OA patients without CS

Table 5 summarizes the results of the logistic regression models that investigate the differential MCMI-III psychopathological profiles of OA-CS *versus* OA-noCS patients. In severe personality pathology subscales, OA-CS patients are more likely to report larger scores only Borderline show significant differences (increase in ORadj between 0.2% and 10.4%). There were no differences in clinical syndrome scales, however, in severe clinical syndrome subscales OA-CS patients report significant differences in Major Depression (increase in ORadj between 0.7% and 9.7%) subscale.

**Table 5:** Differential MCMI-III psychopathological profiles of *OA patients with CS versus OA patients without CS*.

OA-CS *vs* OA-noCS				
		OR_adj_ (95% CI)	(OR_adj_—1) * 100	*p*
Severe Personality Pathology	Bor ^C^	1.052 (1.002–1.104)	5.2% (.2% − 10.4%)	.042
Clinical Syndromes	*Som*	*1*.*043 (*.*994–1*.*094)*	*4*.*3% (-*.*6% − 9*.*4%)*	.*085*
Severe Clinical Syndromes	MDpr ^A^	1.051 (1.007–1.097)	5.1% (.7% − 9.7%)	.023

Logistic regression adjusted Odds ratio (OR_adj_), 95% confidence intervals (95% CI) and p-values (p). (OR_adj_—1) * 100 shows percentage of change. The initial model included gender, age, academic level and cognitive screening.

^C^: only Cognitive Screening included in the final model

^A^: only Age included in the final model. OA-CS: osteoarthritis patients with Central Sensitization; OA-noCS: osteoarthritis patients with Central Sensitization; Bor: borderline; Som: somatoform; MDpr: major depression

### FM patients versus OA patients with CS

Table 6 summarizes the results of the logistic regression models that investigate the differential MCMI-III psychopathological profiles of OA-CS *versus* FM patients. We only found differences in 2 subscales, one from clinical syndromes and another from severe ones. Patients with FM are more likely to report more increased scores in Somatoform (increase in ORadj between 0.2%– 10.5%) subscale. With regard to severe syndromes, Major Depression is more likely to appear in FM patients (increase in ORadj between 1.5% and 11.3%).

**Table 6:** Differential MCMI-III psychopathological profiles of *OA patients with CS versus FM patients*.

FM *vs* OA-CS				
		OR_adj_ (95% CI)	(OR_adj_—1) * 100	*P*
Clinical Personality Patterns	*Dpr*^*N*^	*1*.*053 (*.*994–1*.*115)*	*5*.*3% (-*.*6% − 11*.*5%)*	.*078*
	*Sad*^*N*^	*1*.*058 (*.*995–1*.*125)*	*5*.*8% (-*.*5% − 12*.*5%)*	.*073*
	*Pas*^*N*^	.*946 (*.*892–1*.*003)*	*-5*.*4% (*.*3% − -10*.*8%)*	.*063*
Clinical Syndromes	Som^N^	1.052 (1.002–1.105)	5.2% (.2%– 10.5%)	.043
	*PTSD* ^*N*^	.*938 (*.*878–1*.*003)*	*-6*.*2% (-12*.*2%–*.*3%)*	.*061*
Severe Clinical Syndromes	MDpr^AL^	1.063 (1.015%– 1.113%)	6.3% (1.5%– 11.3%)	.009

Logistic regression adjusted Odds ratio (OR_adj_), 95% confidence intervals (95% CI) and p-values (p). (OR_adj_—1) * 100 shows percentage of change. The initial model included gender, age, academic level and cognitive screening.

^N^: no confounders were included in the final model.

^AL^: only Academic Level included in the final model. Dpr: depressive; Sad: sadistic; Pas: passive-aggressive; Som: somatoform; PTSD: posttraumatic stress disorder; Mdpr: major depression

### OA patients without CS versus FM patients

Table 7 summarizes the results of the logistic regression models that investigate the differential MCMI-III psychopathological profiles of OA-noCS *versus* FM patients. FM patients are more likely to report higher scores in Schizoid (increase in ORadj between 0.8% and 12.5%), Depressive (increase in ORadj between 2.5% and 17.1%), Histrionic (increase in ORadj between 1.4% and 15.3%) and Sadistic (increase in ORadj between 1.7% and 13%) subscales of clinical personality pattern. On severe personality pathology, FM patients are more likely to report higher scores in Borderline subscale (increase in ORadj between 0.2% and 7.2%). OA-noCS patients are more likely to show higher scores in Somatoform scale (increase in ORadj between 1.01% and 11.85%) and lower scores in Posttraumatic stress disorder scale (decrease in ORadj between 0.1% and 14%). In severe clinical syndromes, FM patients are more likely to show increased scores in Major depression scale (increase in ORadj between 3.8% and 12.8%).

**Table 7:** Differential MCMI-III psychopathological profiles of *OA patients without CS versus FM patients*.

FM *vs* OA-noCS				
		OR_adj_ (95% CI)	(OR_adj_—1) * 100	*P*
	Schd^N^	1.065 (1.008–1.1125)	6.5% (.8%-12.5%)	.024
Clinical Personality Patterns	Dpr^N^	1.096 (1.025–1.171)	9.6% (2.5%-17.1%)	.007
	His^N^	1.081 (1.014–1.153)	8.1% (1.4%-15.3%)	.017
	Sad^N^	1.072 (1.017–1.13))	7.2% (1.7%-13%)	.010
	*Msch*^*N*^	*0*.*941 (0*.*883–1*.*002)*	*-5*.*9% (-11*.*7%-*.*2%)*	.*058*
Personality Disorders	Bor^AL^	1.036 (1.002–1.072)	3.6% (.2%-7.2%)	.036
Clinical Syndromes	Som^AL^	1.063 (1.01–1.119)	6.2% (1.01%– 11.85%)	.019
	PTSD^AL^	.927 (.86 –.999)	-7.3% (-14%–-.1%)	.048
Severe Clinical Syndromes	MDpr^AL^	1.082 (1.038–1.128)	8.2% (3.8%– 12.8%)	.000

Logistic regression adjusted Odds ratio (OR_adj_), 95% confidence intervals (95% CI) and p-values (p). (OR_adj_—1) * 100 shows percentage of change. The initial model included gender, age, academic level and cognitive screening.

^N^: no confounders were included in the final model.

^AL^: only Academic Level included in the final model; Schd: schizoid; Dpr: depressive; His: histrionic; Sad: sadistic; Msch: masochistic; Bor: borderline; Som: somatoform; PTSD: posttraumatic stress disorder; MDpr: major depression

## Discussion

**Fig 3:**
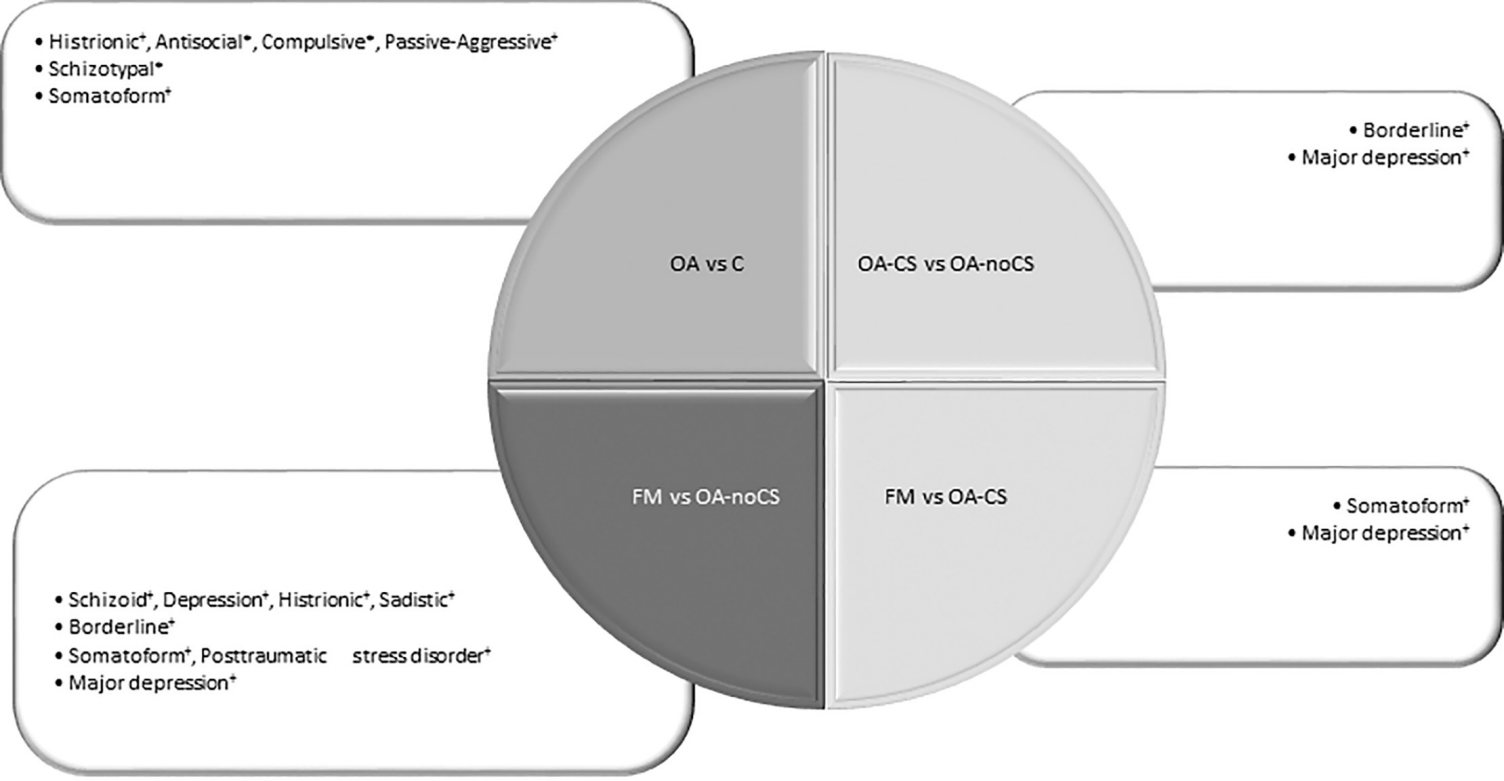
Characteristic subscales of MCMI-III of each group of patients. ^+^ higher scores;*lower scores.

The purposes of this study are to investigate the psychopathological profile of knee OA patients with and without CS, and to compare them with the psychopathological profile of FM patients. In Fig 3 we show the summary of all subscales which conform the different psychopathological profiles.

After divide the subjects depending on the psychometric significance scores (PREV≥75) we realize that the only conditions that are truly related to group of patients are Anxiety, Somatoform, Dysthymia and Major depression. So, patients show mainly emotional alterations or affective problems and theses could be the first target to assess in CP. If we look at the mean scores, contradicting the main evidence in this field at this moment [5], we note that none of the scales showed clinical significance, so there is no presence of psychopathology in any group of patients and none of the personality patterns are clinically relevant. Only Somatoform clinical syndrome show statistical significance in FM group. If we put together the results regarding psychometric significance scores and mean scores, we could think that Dysthymia is a very important disease in CP. Going a little further, we think that it is even more relevant in FM patients, if we focus in the large differences showed in MCMI-III inventory. In fact, Garcia-Fontanals et al. [41] found that 50% of patients with FM showed Dysthymia.

Due to the absence of clinical significance in psychopathological scales the interpretations always will refer to tendency to feel, behave and/or think in a certain way. Besides, this is an exploratory study as we found little evidences in this field, so the comparisons with others studies are quite difficult.

Regarding OA profile (without taking account if there is CS or not) versus Control subjects, there is a clear and differentiated profile. Patients are more likely to score higher in Histrionic and Passive-Aggressive, and lower in Antisocial and Compulsive clinical personality patterns scales. They also rate lower in Schizotypal personality scale. In clinical syndromes, the score of Somatoform scale is more likely to be higher. So, we could say that patients with OA are more likely to show gregarious self-image, dramatic speech, being interpersonally submissive, irritability, express resentful, felling blamed and shamed, and show physical weakness, fatigue, exaggeration of physical symptoms and health worries. On the other hand, insensitivity with others, suspicion, being demanding and perfectionist, social isolation and eccentricity are not distinctive features. Thus, the combination of positive and negative emotions [4] and attention-seeking compose a brief overview of OA patients.

The second comparison shows the characteristic features of FM versus OA-noCS. In this case, FM profile is composed by schizoid, depression, histrionic and sadistic patterns of personality, borderline personality disorder, somatoform and posttraumatic stress disorder and major depression as clinical syndromes. So, they are more likely to reflect apathy, lack of pleasure, pessimism, hopelessness, need of affection, avoidance of disapproval, covert hostility, emotional lability, expression of psychological distress trough physical and health complaints and worries, trauma-related emotions associated to anxious activation and avoidance of the environment of that stressful event and devaluation, feelings of blame, food alterations, sleep alterations, depreciation and lack of concentration and motivation [42]. So, in summary, it is related to isolation, exaggeration and feelings of anxiety and sadness.

The third profile is to differentiate OA-CS from OA-noCS where OA-CS patients are characterized for borderline and major depression tendency. These scales are related to being temperamentally labile, combination of anger and sadness, uncertain self-image or identity, anxiety, and hopelessness, apathy, psychomotor delay or agitation, problems of sleep, food, weight, cognitive alterations like attention and concentration and feelings of blame. So, the profile focuses on emotional alterations [5].

And finally, the comparison between both groups with CS, FM and OA-CS, is composed by somatoform and major depression traits. That means, somatic complaints due to emotional discomfort, tiredness, weakness, worry about health, disease oversize and feelings of indifference, desperation, psychomotor agitation or delay, sleep and food difficulties, weight loss or gain, concentration alterations and guilt. In sum, mood alterations and health complaints are more likely to appear in FM patients.

As found in previous works regarding FM patients [18], our profile characteristics address to anxiety and depression. However, there are various types of anxiety and depression and they should be differentiated to offer better health care. Anxiety is defined as phobic, tense, restless, undecided, excessive transpiration, digestive alterations, wet hands, easily becoming startled and alertness. Though, somatoform and posttraumatic stress disorder are not the same even they are anxiety syndromes. As we mentioned above, somatoform pattern is characterized by tiredness and weakness periods, concerns over the health, non-specific pain and sensations with not necessary related to primary disease (FM or OA, in our case). Posttraumatic stress condition consists in having suffered an event considered threatening (could be the diagnosis of a disease) and react to it with fear and helplessness, images, emotions, memories and thoughts related to the event, hypervigilance and being startled.

Beyond the specific mood alterations that FM and OA patients actually show, this study also takes into account coping styles and certain personality disorders. In periods of stress, the traits intensity rises and cause more maladaptive behaviors than in normal and routine situations. This fact implies an increase of strength of psychological symptoms (in this case, somatoform and posttraumatic stress). Characteristic behaviors are dramatic, emotional, attention-seeking and fluctuating mood for all patients, however, FM use to show higher scores.

Our study has some limitations: 1) the sample is reduced, especially for OA-CS group, because of the accuracy in inclusion/exclusion criteria; 2) Although a conservative cut-off score of 75 or more for each of the 10 clinical syndrome scales has been established as a criterion for the presence of symptomatology clinically relevant, the likelihood of Type II error due to the small sample size of our study groups may have hidden other differences in the differential MCMI-III psychopathological profiles analysed; 3) in FM group there are only women, with high educations levels and most of them were taking antidepressants, so it is challenging to generalize the results.

Hence, this study highlights in the differentiated pattern of FM and OA-CS groups, both with CS, which is quite specific and focus on somatic symptoms, guilty feelings, resignation, agitation or delay and ruminative thoughts, mainly. The strategies to affront illness in patients with CS are the same. It is not clear if there is a specific psychopathological profile for CS syndromes, but FM and OA-CS are more similar than with OA-noCS, and they only show differences in somatic symptoms and mood.

With a detailed and comprehensive study of the psychopathological characteristics of patients suffering from CP, a more precise and rich description is obtained that will help a better description of the patient and consequently the treatment may also be more individualized [42]. Clinicians may could consider not only pharmacological treatments, but also cognitive behavioral therapy and aerobic exercise, in some cases, as the first choice treatment [43], including biopsychosocial perspective as a very effective conceptualization for treatment [44]. Patients with OA, with and without CS, need to be considered differently by all health workers because it is clear that suffering from CP involve complex personality and psychological patterns [45] and the fact of suffering a very common disease should not translate to being normal and expected, but very important because affects a large amount of people.

In conclusion, we found that presence of CS in OA patients is related to larger presence of psychopathology. So, perhaps, CS is a risk factor to suffer more psychopathological symptoms, which brings to a more complicated clinical prognostic of these patients, as Galvez-Sánchez Duschek and Reyes del Paso found [46]. This is the reason why we think our study could be interesting for all health professionals, including disciplines of rheumatology, internal medicine, psychiatry, primary care physician, and neurology, among others.

Further studies are necessary to replicate, or not, our findings and to fully investigate the whole psychopathological profile of OA, FM and CS syndromes. Maybe this investigation encourages researchers to look for new ways to treat OA, FM and CS patients and they can have less pain and better quality of life.

## References

[1] Peat G, McCarney R, Croft P. Knee pain and osteoarthritis in older adults: a review of community burden and current use of primary health care. Ann Rheum Dis. 2001;60: 91–97. 10.1136/ard.60.2.91 11156538 PMC1753462

[2] Queiroz LP. Worldwide epidemiology of fibromyalgia. Curr Pain Headache Rep. 2013;17: 356 10.1007/s11916-013-0356-5 23801009

[3] Johnson VL, Hunter DJ. The epidemiology of osteoarthritis. Best Pract Res Clin Rheumatol. 2014;28: 5–15. 10.1016/j.berh.2014.01.004 24792942

[4] Axford J, Butt A, Heron C, Hammond J, Morgan J, Alavi A, et al Prevalence of anxiety and depression in osteoarthritis: use of the Hospital Anxiety and Depression Scale as a screening tool. Clin Rheumatol. 2010;29: 1277–1283. 10.1007/s10067-010-1547-7 20721594

[5] Huang S-W, Wang W-T, Lin L-F, Liao C-D, Liou T-H, Lin H-W. Association between psychiatric disorders and osteoarthritis: a nationwide longitudinal population-based study. Medicine (Baltimore). 2016;95: e4016 10.1097/MD.0000000000004016 27368019 PMC4937933

[6] Kittelson AJ, George SZ, Maluf KS, Stevens-Lapsley JE. Future directions in painful knee osteoarthritis: harnessing complexity in a heterogeneous population. Phys Ther. 2014;94: 422–432. 10.2522/ptj.20130256 24179141 PMC3967122

[7] Helminen E-E, Sinikallio SH, Valjakka AL, Väisänen-Rouvali RH, Arokoski JP. Determinants of pain and functioning in knee osteoarthritis: a one-year prospective study. Clin Rehabil. 2016;30: 890–900. 10.1177/0269215515619660 27496698 PMC4976658

[8] Riddle DL, Kong X, Fitzgerald GK. Psychological Health Impact on Two-Year Changes in Pain and Function in Persons with Knee Pain: Data from the Osteoarthritis Initiative. Osteoarthr Cartil OARS Osteoarthr Res Soc. 2011;19: 1095–1101. 10.1016/j.joca.2011.06.003 21723400 PMC3159740

[9] Stubbs B, Aluko Y, Myint PK, Smith TO. Prevalence of depressive symptoms and anxiety in osteoarthritis: a systematic review and meta-analysis. Age Ageing. 2016;45: 228–235. 10.1093/ageing/afw001 26795974

[10] Perrot S. Osteoarthritis pain. Best Pract Res Clin Rheumatol. 2015;29: 90–97. 10.1016/j.berh.2015.04.017 26267003

[11] Wolfe F, Smythe HA, Yunus MB, Bennett RM, Bombardier C, Goldenberg DL, et al The American College of Rheumatology 1990 Criteria for the Classification of Fibromyalgia. Report of the Multicenter Criteria Committee. Arthritis Rheum. 1990;33: 160–172. 2306288 10.1002/art.1780330203

[12] Wolfe F, Clauw DJ, Fitzcharles M-A, Goldenberg DL, Katz RS, Mease P, et al The American College of Rheumatology preliminary diagnostic criteria for fibromyalgia and measurement of symptom severity. Arthritis Care Res. 2010;62: 600–610. 10.1002/acr.20140 20461783

[13] Meeus M, Nijs J. Central sensitization: a biopsychosocial explanation for chronic widespread pain in patients with fibromyalgia and chronic fatigue syndrome. Clin Rheumatol. 2007;26: 465–473. 10.1007/s10067-006-0433-9 17115100 PMC1820749

[14] Sluka KA, Clauw DJ. Neurobiology of fibromyalgia and chronic widespread pain. Neuroscience. 2016;338: 114–129. 10.1016/j.neuroscience.2016.06.006 27291641 PMC5083139

[15] Huber A, Suman AL, Biasi G, Carli G. Predictors of psychological distress and well-being in women with chronic musculoskeletal pain: two sides of the same coin? J Psychosom Res. 2008;64: 169–175. 10.1016/j.jpsychores.2007.09.005 18222130

[16] Epstein SA, Kay G, Clauw D, Heaton R, Klein D, Krupp L, et al Psychiatric disorders in patients with fibromyalgia. A multicenter investigation. Psychosomatics. 1999;40: 57–63. 10.1016/S0033-3182(99)71272-7 9989122

[17] Dobkin PL, Civita MD, Abrahamowicz M, Baron M, Bernatsky S. Predictors of health status in women with fibromyalgia: A prospective study. Int J Behav Med. 2006;13: 101–108. 10.1207/s15327558ijbm1302_1 16712427

[18] Fietta P, Fietta P, Manganelli P. Fibromyalgia and psychiatric disorders. Acta Bio-Medica Atenei Parm. 2007;78: 88–95. 17933276

[19] Yunus MB. Fibromyalgia and overlapping disorders: the unifying concept of central sensitivity syndromes. Semin Arthritis Rheum. 2007;36: 339–356. 10.1016/j.semarthrit.2006.12.009 17350675

[20] Aaron LA, Bradley LA, Alarcón GS, Alexander RW, Triana-Alexander M, Martin MY, et al Psychiatric diagnoses in patients with fibromyalgia are related to health care-seeking behavior rather than to illness. Arthritis Rheum. 1996;39: 436–445. 10.1002/art.1780390311 8607892

[21] Giesecke T, Williams DA, Harris RE, Cupps TR, Tian X, Tian TX, et al Subgrouping of fibromyalgia patients on the basis of pressure-pain thresholds and psychological factors. Arthritis Rheum. 2003;48: 2916–2922. 10.1002/art.11272 14558098

[22] Jensen KB, Petzke F, Carville S, Fransson P, Marcus H, Williams SCR, et al Anxiety and depressive symptoms in fibromyalgia are related to poor perception of health but not to pain sensitivity or cerebral processing of pain. Arthritis Rheum. 2010;62: 3488–95. 10.1002/art.27649 20617526

[23] de Souza JB, Potvin S, Goffaux P, Charest J, Marchand S. The deficit of pain inhibition in fibromyalgia is more pronounced in patients with comorbid depressive symptoms. Clin J Pain. 2009;25: 123–127. 10.1097/AJP.0b013e318183cfa4 19333157

[24] van Wilgen CP, Keizer D. The sensitization model to explain how chronic pain exists without tissue damage. Pain Manag Nurs Off J Am Soc Pain Manag Nurses. 2012;13: 60–65. 10.1016/j.pmn.2010.03.001 22341140

[25] Woolf CJ. Central sensitization: implications for the diagnosis and treatment of pain. Pain. 2011;152: S2–15. 10.1016/j.pain.2010.09.030 20961685 PMC3268359

[26] Lluch E, Torres R, Nijs J, Van Oosterwijck J. Evidence for central sensitization in patients with osteoarthritis pain: A systematic literature review. Eur J Pain. 2014;18: 1367–1375. 10.1002/j.1532-2149.2014.499.x 24700605

[27] Lluch Girbés E, Dueñas L, Barbero M, Falla D, Baert IAC, Meeus M, et al Expanded Distribution of Pain as a Sign of Central Sensitization in Individuals With Symptomatic Knee Osteoarthritis. Phys Ther. 2016;96: 1196–1207. 10.2522/ptj.20150492 26939604

[28] Wood LRJ, Peat G, Thomas E, Duncan R. Knee osteoarthritis in community-dwelling older adults: are there characteristic patterns of pain location? Osteoarthritis Cartilage. 2007;15: 615–623. 10.1016/j.joca.2006.12.001 17276094

[29] Finan PH, Buenaver LF, Bounds SC, Hussain S, Park RJ, Haque UJ, et al Discordance between pain and radiographic severity in knee osteoarthritis: findings from quantitative sensory testing of central sensitization. Arthritis Rheum. 2013;65: 363–372. 10.1002/art.34646 22961435 PMC3863776

[30] George S, Wittmer V, Fillingim R, Robinson M. Psychological influence on central sensitization of pain for patients with chronic low back pain. J Pain. 2005;6: S83 10.1016/j.jpain.2005.01.328

[31] Imamura M, Imamura ST, Kaziyama HHS, Targino RA, Hsing WT, de Souza LPM, et al Impact of nervous system hyperalgesia on pain, disability, and quality of life in patients with knee osteoarthritis: a controlled analysis. Arthritis Rheum. 2008;59: 1424–1431. 10.1002/art.24120 18821657

[32] Fleming KC, Volcheck MM. Central sensitization syndrome and the initial evaluation of a patient with fibromyalgia: a review. Rambam Maimonides Med J. 2015;6: e0020 10.5041/RMMJ.10204 25973272 PMC4422459

[33] Arendt-Nielsen L, Nie H, Laursen MB, Laursen BS, Madeleine P, Simonsen OH, et al Sensitization in patients with painful knee osteoarthritis. Pain. 2010;149: 573–581. 10.1016/j.pain.2010.04.003 20418016

[34] Altman R, Asch E, Bloch D, Bole G, Borenstein D, Brandt K, et al Development of criteria for the classification and reporting of osteoarthritis. Classification of osteoarthritis of the knee. Diagnostic and Therapeutic Criteria Committee of the American Rheumatism Association. Arthritis Rheum. 1986;29: 1039–1049. 10.1002/art.1780290816 3741515

[35] Millon T, Millon C, Davis R, Grossman S. MCMI-III: Millon Clinical Multiaxial Inventory. Pearson 2009.

[36] Burckhardt CS, Clark SR, Bennett RM. The fibromyalgia impact questionnaire: development and validation. J Rheumatol. 1991;18: 728–733. 1865419

[37] Monterde S, Salvat I, Montull S, Fernández-Ballart J. Validación de la versión española del Fibromyalgia Impact Questionnaire. Rev Esp Reumatol. 2004;31: 507–513.

[38] Bellamy N, Buchanan WW, Goldsmith CH, Campbell J, Stitt LW. Validation study of WOMAC: a health status instrument for measuring clinically important patient relevant outcomes to antirheumatic drug therapy in patients with osteoarthritis of the hip or knee. J Rheumatol. 1988;15: 1833–1840. 3068365

[39] Escobar A, Quintana JM, Bilbao A, Azkárate J, Güenaga JI. Validation of the Spanish version of the WOMAC questionnaire for patients with hip or knee osteoarthritis. Western Ontario and McMaster Universities Osteoarthritis Index. Clin Rheumatol. 2002;21: 466–471. 10.1007/s100670200117 12447629

[40] Villanueva I, del Mar Guzman M, Javier Toyos F, Ariza-Ariza R, Navarro F. Relative efficiency and validity properties of a visual analogue vs a categorical scaled version of the Western Ontario and McMaster Universities Osteoarthritis (WOMAC) Index: Spanish versions. Osteoarthritis Cartilage. 2004;12: 225–231. 10.1016/j.joca.2003.11.006 14972339

[41] Garcia-Fontanals A, Portell M, García-Blanco S, Poca-Dias V, García-Fructuoso F, López-Ruiz M, et al Vulnerability to Psychopathology and Dimensions of Personality in Patients With Fibromyalgia. Clin J Pain. 2017;33: 991–997. 10.1097/AJP.0000000000000506 28448425

[42] Uçar M, Sarp Ü, Karaaslan Ö, Gül AI, Tanik N, Arik HO. Health anxiety and depression in patients with fibromyalgia syndrome. J Int Med Res. 2015;43: 679–685. 10.1177/0300060515587578 26249741

[43] Häuser W, Fitzcharles M-A. Facts and myths pertaining to fibromyalgia. Dialogues Clin Neurosci. 2018;20: 53–62. 29946212 10.31887/DCNS.2018.20.1/whauser PMC6016048

[44] Adams LM, Turk DC. Psychosocial Factors and Central Sensitivity Syndromes. Curr Rheumatol Rev. 2015;11: 96–108. 10.2174/1573397111666150619095330 26088211 PMC4728142

[45] Wise B, Niu J, Zhang Y, Wang N, Jordan J, Choy E, et al Psychological factors and their relation to osteoarthritis pain. Osteoarthr Cartil OARS Osteoarthr Res Soc. 2010;18: 883–887. 10.1016/j.joca.2009.11.016 20346403 PMC2912218

[46] Galvez-Sánchez CM, Duschek S, Reyes del Paso GA. Psychological impact of fibromyalgia: current perspectives. Psychol Res Behav Manag. 2019;12: 117–127. 10.2147/PRBM.S178240 30858740 PMC6386210

